# Oxygen Restriction Generates Difficult-to-Culture *P. aeruginosa*

**DOI:** 10.3389/fmicb.2019.01992

**Published:** 2019-08-29

**Authors:** Lasse Kvich, Blaine Fritz, Stephanie Crone, Kasper N. Kragh, Mette Kolpen, Majken Sønderholm, Mikael Andersson, Anders Koch, Peter Ø. Jensen, Thomas Bjarnsholt

**Affiliations:** ^1^Costerton Biofilm Center, Department of Immunology and Microbiology, Faculty of Health and Medical Sciences, University of Copenhagen, Copenhagen, Denmark; ^2^Department of Clinical Microbiology, Copenhagen University Hospital – Rigshospitalet, Copenhagen, Denmark; ^3^Department of Epidemiology Research, Statens Serum Institut, Copenhagen, Denmark; ^4^Department of Infectious Diseases, Copenhagen University Hospital – Rigshospitalet, Copenhagen, Denmark

**Keywords:** *P. aeruginosa*, *S. aureus*, *S. epidermidis*, anoxia, biofilm, reactive oxygen species, difficult-to-culture, viable but non-culturable

## Abstract

Induction of a non-culturable state has been demonstrated for many bacteria, e.g., *Escherichia coli* and various *Vibrio* spp. In a clinical perspective, the lack of growth due to these non-culturable bacteria can have major consequences for the treatment of patients. Here, we show how anoxic conditioning (restriction of molecular oxygen, O_2_) generates difficult-to-culture (DTC) bacteria during biofilm growth. A significant subpopulation of *Pseudomonas aeruginosa* entered a DTC state after anoxic conditioning, ranging from 5 to 90% of the total culturable population, in both planktonic and biofilm models. Anoxic conditioning also generated DTC subpopulations of *Staphylococcus aureus* and *Staphylococcus epidermidis* (89 and 42% of the total culturable population, respectively). Growth of the DTC populations were achieved by substituting O_2_ with 10 mM NO_3_^–^ as an alternative electron acceptor for anaerobic respiration or, in the case of *P. aeruginosa*, by adding sodium pyruvate or catalase as scavengers against reactive oxygen species (ROS) during aerobic respiration. An increase in normoxic plating due to addition of catalase suggests the molecule hydrogen peroxide as a possible mechanism for induction of DTC *P. aeruginosa.* Anoxic conditioning also generated a true viable but non-culturable (VBNC) population of *P. aeruginosa* that was not resurrected by substituting O_2_ with NO_3_^–^ during anaerobic respiration. These results demonstrate that habituation to an anoxic micro-environment could complicate diagnostic culturing of bacteria, especially in the case of chronic infections where oxygen is restricted due to the host immune response.

## Introduction

Clinical laboratories often culture bacteria with enriched media, developed to support maximal growth of particular pathogens. However, even these bacterial human pathogens can enter a growth-restricted, transient state resulting in loss of culturability, especially following antibiotic treatment (Pasquaroli et al., 2013). Non-culturable bacteria are apparent by microscopic identification in clinical cases presenting sign of infection, but no positive cultures. These cases are especially prevalent during chronic infection, where lack of culturability prevents proper diagnosis and treatment (Høiby et al., 2010, 2015; Costerton et al., 2011).

Two well defined, yet difficult to distinguish, non-culturable states have been described for bacteria: viable but non-culturable (VBNC) and persisters (Oliver, 2010; Wood et al., 2013). Persisters are described as growth-arrested subpopulations of transiently antibiotic-tolerant cells that arise in a population after antibiotic treatment (Fisher et al., 2017). VBNC cells are defined by their lack of growth during conventional plating, though they remain viable and potentially virulent. The VBNC state is induced by a variety of stresses and removing these stresses can often resuscitate the VBNC cells, facilitating growth (Oliver, 2010). Recent evidence shows that these two states share many similarities (Ayrapetyan et al., 2015, 2018) and some even suggest that they describe the same state (Kim et al., 2018). VBNC cells are, by definition, in a state where they are no longer culturable and alternative methods, which are not based on cultivation, are necessary to detect cells in this state. Therefore, an alternate definition is necessary to describe cells that are resuscitated from a non-culturable state. In this paper, we term this state as difficult-to-culture (DTC).

Several environmental stresses have been associated with non-culturable states, e.g., oxidative stress, change in temperature or pH, nutrient starvation, change in osmotic concentrations and presence of heavy metals or antibiotics (Oliver, 2010; Ayrapetyan et al., 2015). Oxygen starvation, referred to as anoxic conditioning in this paper, has also shown to induce a non-culturable state in batch cultures of *Pseudomonas aeruginosa*, but supplementation with nitrate (NO_3_^–^) as an alternative electron acceptor during anoxic plating restored culturability (Binnerup and Sørensen, 1993). The lack of growth during normoxic plating suggests that anoxic conditioning sensitizes a subpopulation of *P. aeruginosa* to O_2_ or its toxic derivatives, such as reactive oxygen species (ROS). ROS are continuously created during aerobic respiration by incomplete reduction of O_2__,_ which is toxic to cells if not scavenged (Fenchel and Finlay, 2008). Presence of ROS has, in some studies, been shown to generate VBNC bacteria (Oliver, 2010; Noor, 2015), but most research has focused on *Escherichia coli* strains or *Vibrio* spp., while the effects of ROS on *P. aeruginosa* and other facultative pathogens are not well characterized.

*Pseudomonas aeruginosa* is an opportunistic pathogen isolated from both acute and chronic infections (Turner et al., 2014). The role of *P. aeruginosa* in chronic infections is best described for cystic fibrosis (CF), where it is the predominant cause of morbidity and mortality (Candido Cacador et al., 2018). Establishment of a biofilm in the CF lungs with a mucoid phenotype of *P. aeruginosa* is a chronic condition that requires lifetime-treatment with antimicrobial therapy (Bjarnsholt et al., 2009).

Evidence of anoxic zones and anaerobic bacterial activity in chronic infections suggests that colonizing bacteria, such as *P. aeruginosa*, experience anoxic conditioning (Hassett et al., 2002; Worlitzsch et al., 2002; Kolpen et al., 2014). High rates of O_2_ consumption by polymorphonuclear leukocytes generate local, anoxic zones and likely play a major role in O_2_ depletion during chronic infection (Høiby et al., 2015). In addition, other cells of the host may also consume O_2_, increasing the likelihood of bacteria experiencing anoxia (Worlitzsch et al., 2002). Many chronic infections are thought to contain bacteria in the biofilm mode of growth (Costerton et al., 2003) and endogenous O_2_ depletion inside the biofilm (Sønderholm et al., 2018), along with intense O_2_ consumption by the host immune response (Worlitzsch et al., 2002; Kolpen et al., 2010; Trunk et al., 2010; Kragh et al., 2014; James et al., 2016; Jensen et al., 2017), may therefore increase the number of VBNC bacteria.

This study aimed to determine whether anoxic conditioning generates VBNC cells of *P. aeruginosa* and if anoxic conditioning induces a DTC state during biofilm growth. Additionally, we investigated whether ROS are involved in the loss of culturability and if non-culturable populations of other facultative human pathogens could be induced by anoxic conditioning when grown as biofilm.

## Materials and Methods

### Bacterial Strains

*Pseudomonas aeruginosa* (PAO1, ATCC 15692), a catalase A deficient *Pseudomonas aeruginosa* strain (Δ*katA* PAO1) (Hassett et al., 1999), *Staphylococcus epidermidis* ATCC 14990, *Staphylococcus aureus* NCTC 8325-4 (methicillin susceptible) (Frees et al., 2004), *S. aureus* USA300 JE2 (MRSA) (Fey et al., 2013), *E. coli* CFT073 (Welch et al., 2002) and a clinical strain of *Enterococcus faecalis* from the Department of Clinical Microbiology, Copenhagen University Hospital – Rigshospitalet, Denmark were used in this study.

### Agar Plates and Media

Primarily lysogeny broth (LB) agar plates were applied in this study. LB (pH 7.5) consisted of 5 g/L yeast extract (Oxoid, Roskilde, Denmark), 10 g/L tryptone (Oxoid), 10 g/L NaCl (Merck, United States). All plates contained 2% agar. Plates used for anoxic growth were supplemented with 10 mM KNO_3_ (Sigma-Aldrich, United States) to serve as alternative electron acceptor during anoxic plating, referred to as NO_3_^–^ throughout the paper. All agar plates and media in this study were supplied by the Panum Institute Substrate Department (Copenhagen, Denmark).

### Anoxic Growth

Experiments investigating growth under anoxic conditions were performed in an anaerobic chamber (Concept 400 Anaerobic Workstation, Ruskinn Technology Ltd., United Kingdom). The gas atmosphere consisted of N_2_/H_2_/CO_2_ (ratio – 80:10:10). Anoxic chamber environment was confirmed with an oxygen sensor (HQ40d multi, HACH Company, United States). All media and chemical solutions used in anaerobic experiments were equilibrated in the anaerobic chamber 3 days prior to experiment. In the case of solutions requiring refrigeration, a minimal volume was applied (<1 mL), sealed with Parafilm M, and thoroughly shaken upon entry into anaerobic chamber for quick gas equilibration.

### Colony Forming Units per Milliliter (CFU/mL)

**FIGURE 1:**
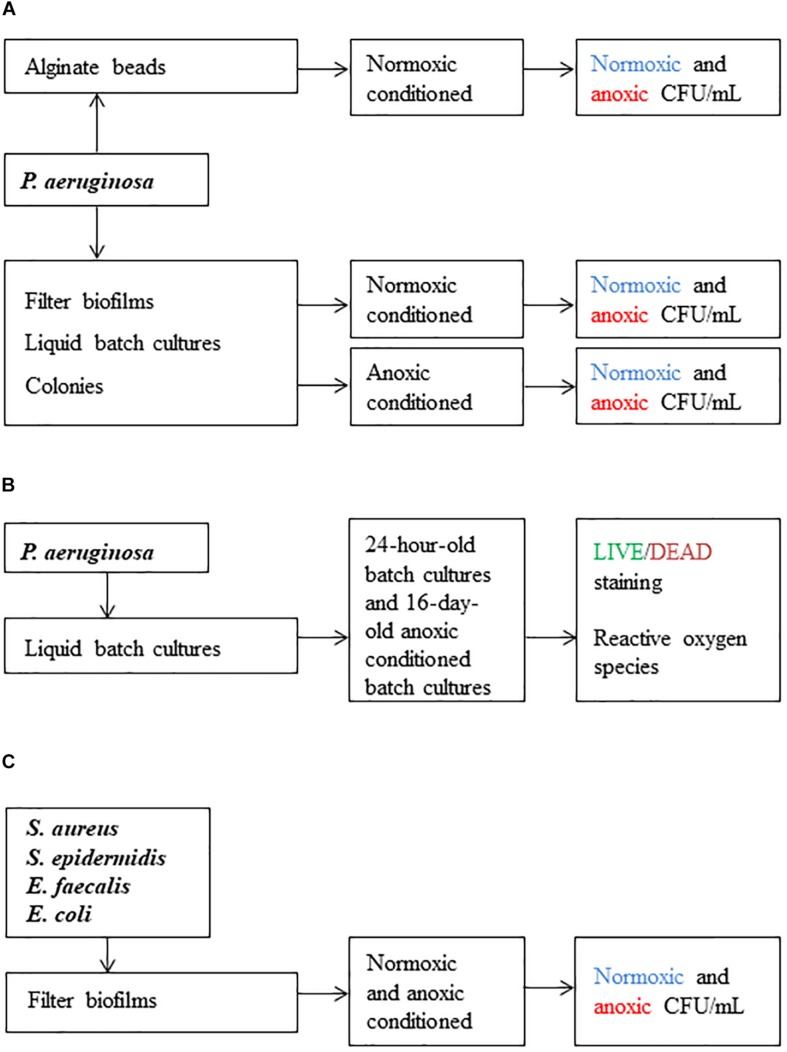
Scheme of the workflow used in this study. Methods applied in **(A)** were used to investigate difficult-to-culture (DTC) subpopulations. Methods applied in **(B)** were used to determine viable but-non-culturable (VBNC) subpopulations and the effect of reactive oxygen species (ROS). Methods applied in **(C)** were used to investigate if other pathogens were sensitive to anoxic conditioning. Colony forming units per milliliter (CFU/mL) was the main outcome.

CFU/mL was the main outcome in this study. We demonstrate the log-difference in CFU/mL between types of incubation (normoxic vs. anoxic) after bacteria had been grown with and without oxygen, respectively. An overview of the workflow is presented in Figure 1. Bacterial suspensions were sonicated in an ultrasonic water bath (5 min degas + 5 min sonication; Bransonic ultrasonic cleaner 2510, Emerson Electric, United States) before ten-fold dilution series were performed in 0.9% NaCl. CFU was determined by plating 3 × 10 μL-drops per dilution per replicate. Anoxic CFU determinations were performed inside an anaerobic chamber on LB plates supplemented with 10 mM NO_3_^–^, unless other is stated. The same dilution series were applied to normoxic CFU determination outside the anaerobic chamber on LB plates with 10 mM NO_3_^–^, unless other stated. Anoxic plates were incubated 2 days at 37°C in the anaerobic chamber before counting CFU. Normoxic plates were likewise incubated for 2 days, but, after 24 h growth at 37°C, plates were incubated for an additional 24 h at room temperature to avoid overgrowth that would bias or make counting of CFU difficult.

### Bead-Embedded Inoculum of *P. aeruginosa*

Preparation of alginate beads with *P. aeruginosa* was carried out according to a method described by Sønderholm et al. (2017). Subsequently, beads were divided (10 beads per vial, 1 vial per sampling day) into vials (Oximate Vial, PerkinElmer Inc., United States) containing 15 mL LB medium. Vials were sealed with Parafilm M (oxygen permeable) and incubated at 37°C in an orbital shaker at 180 rpm. Anoxic and normoxic CFU counts were determined from beads and from the surrounding suspension from the same vials on each sampling day (day 3, 6, 8, 12, 19 and 21). Two beads were sampled per biological replicate (*n* = 4). Before determination of CFU, the beads were washed twice with 0.9% NaCl to remove non-attached cells and transferred to 1.5 mL microcentrifuge tubes (Sigma-Aldrich, United States). One-hundred microliters of 0.1 M sodium carbonate (Na_2_CO_3_) followed by 100 μL of 0.04 M citric acid was added to the tubes to dissolve the beads. When determining CFU from the surrounding suspension, a volume of 1 mL was used. CFU/mL was determined as previously described.

### Filter Biofilms With *P. aeruginosa*

This protocol was adapted to grow reproducible biofilms under anoxic and normoxic conditions. The method has previously been described (Bjarnsholt et al., 2015). The filter biofilms were kept on the same LB plates throughout the experiment. *P. aeruginosa* was propagated from frozen stock and grown overnight in 20 mL LB medium at 37°C in an orbital shaker at180 rpm. Cultures were adjusted to an optical density (OD_600_ nm) of 0.05 (UV spectrophotometer UV-1800 UV-VIS, Shimadzu corporation, JP) and 10 μL was transferred to the cellulose nitrate membrane filters (25 mm in diameter, GE Healthcare Life Sciences, United Kingdom). Plates were incubated under normoxic and anoxic conditions and kept in plastic bags with wet paper to avoid dehydration. Two filters were sampled per biological replicate (*n* = 4) on each sampling day (day 1, 3, 7, 15 and 17). Filters were removed, placed in 10 mL tubes containing 5 mL 0.9% NaCl and vortexed thoroughly (1 min) prior to CFU determination. CFU/mL was determined as previously described.

### Liquid Batch Cultures of *P. aeruginosa*

*Pseudomonas aeruginosa* was propagated from frozen stock and grown overnight in 20 mL LB media at 37^o^C and orbitally shaken at 180 rpm. Cultures were OD adjusted to 0.1 (OD_600_ nm) in glass vials (Oximate Vial) with a final volume of 20 mL LB. Vials were left to incubate at 37°C and orbitally shaken at 180 rpm. To create a normoxic environment, half of the vials were incubated with Parafilm M on top (normoxic conditioning), while the rest were incubated with a lid on top creating an anoxic environment (anoxic conditioning). Anoxic and normoxic plating was carried out from normoxic and anoxic conditioned liquid batch cultures (referred to as batch cultures throughout the paper) on each sampling day (day 1, 3, 5, 9, 11, 14, 16, 18, 21 and 28). Normoxic plating was carried out on LB plates with and without 10 mM NO_3_^–^ to test whether presence of NO_3_^–^ affected the number of CFU. Two mL (2 × 1 mL = 2 technical replicates) were sampled per biological replicate (*n* = 4) on each sampling day. CFU/mL was determined as previously described. The largest log-difference between types of incubation in the liquid batch culture setup was observed at day 16, so this time point and method was selected for further analysis.

### Colonies of *P. aeruginosa*

*Pseudomonas aeruginosa* was propagated from frozen stock and grown overnight in 20 mL LB media at 37^o^C in an orbital shaker at 180 rpm. The cultures were then streaked onto LB plates supplemented with 10 mM NO_3_^–^. Plates were incubated under normoxic and anoxic conditions and kept in plastic bags with wet paper to avoid dehydration. A 1-μL loop was used to sample colony material from each biological replicate (*n* = 3) on each sampling day (day 6, 9, 13, 15 and 20). Colonies were transferred to 1.5 mL microcentrifuge tubes (Sigma-Aldrich, Denmark) with 0.5 mL 0.9% NaCl and vortexed thoroughly. CFU/mL was determined as previously described.

### Direct Viable Count With LIVE/DEAD Staining

LIVE/DEAD staining was applied to estimate the proportion of viable and non-viable cells in 24-hour-old batch cultures and in 16-day-old anoxically conditioned batch cultures of *P. aeruginosa*. Batch cultures were prepared as previously described. The dyes for LIVE/DEAD staining consisted of two fluorescent nucleic acid stains, the green fluorescent stain (live cells) SYTO9 (Invitrogen, United States) and the red fluorescent stain (dead cells) propidium iodide (PI, Sigma-Aldrich, United States). SYTO9 penetrates both intact and damaged membranes while PI only stains damaged cells, thereby creating an opportunity to discriminate between live and dead cells (Li et al., 2014). Bacterial suspensions were vortexed thoroughly (1 min) and sonicated in an ultrasonic water bath (5 min degas + 5 min sonication; Bransonic ultrasonic cleaner 2510, Emerson Electric, United States) before staining. To stain the cells, 1 μL of PI (1 mg/mL) and SYTO9 (5 mM) was added to 1 mL of bacterial suspension and incubated for 15 min at room temperature. Suspensions of each biological replicate were then filtered through a 0.2 μm black Whatman, Nuclepore Trach-Etch Membrane (Sigma-Aldrich, Denmark) and visualized with confocal laser scanning microscopy using a Zeiss LSM 880with a 63 × /1.4 (numerical aperture) objective (Zeiss, Germany). Sequential scanning with individual lasers was applied to detect fluorescence from each fluorophores. SYTO9 was detected with an excitation wavelength of 488 nm and an emission range of 493–578 nm, while PI was detected with an excitation wavelength of 561 nm and an emission range of 578–718. Fifteen random fields (135 μm × 135 μm) were examined for each filter (*n* = 3). Enumeration of live (green) and dead (red) bacteria were done with the IMARIS software package (Bitplane AG, Schwitzerland). Briefly, individual particles in each field were identified and counted as bacteria. Particles that were stained with both PI and SYTO9 were considered non-viable and thus counted as dead cells. Conversion of enumerated viable and dead cells to bacterial counts per milliliter was performed as described (Boulos et al., 1999) to compare them with CFU/mL. Briefly, the numbers of particles per mL were estimated using the following equation$\frac{p\,a\,r\,t\,i\,c\,l\,e\,s}{m\,L}=A^{*}\,\left(\frac{B}{C}\right)/D$. Where A is the amount of particles per field, B is the surface of filtration (mm), C is the area of the microscopic field and D the volume of the sample filtered. Normoxic and anoxic CFU/mL was carried out simultaneously to estimate the proportion of VBNC cells. CFU/mL was determined as previously described.

### Reactive Oxygen Species

To elucidate whether growth of anoxically conditioned bacteria was restricted by creation of ROS during aerobic respiration, sodium pyruvate or catalase (Sigma-Aldrich, United States) was tested as ROS scavenger in LB plates with 10 mM NO_3_^–^. Sodium pyruvate (0.3%) was tested during anoxic and normoxic plating for 24-hour-old batch cultures and anoxically conditioned 16-day-old batch cultures of *P. aeruginosa*. Catalase (∼50,000 units of catalase per L medium) was tested during anoxic and normoxic plating for anoxically conditioned 16-day-old batch cultures of *P. aeruginosa*. Furthermore, CFU/mL was also determined from 16-day-old anoxically conditioned batch cultures of *P. aeruginosa* and a catalase A deficient *P. aeruginosa* (Δ*katA* PAO1) to test the influence of ROS. Lastly, creation of ROS was measured along with OD at day 1, 8 and 16 for normoxically and anoxically conditioned batch cultures of *P. aeruginosa*. ROS production was measured by adding 5 μM 2′,7′dichlorodihydrofluorescein diacetate (DCFH-DA; Sigma-Aldrich, United States) to bacterial suspensions with an OD_600_ nm previously adjusted, in fresh LB medium, to 0.1. This concentration has previously been used to detect ROS for *P. aeruginosa* (Jensen et al., 2014). Two hundred microliter of this suspension were transferred into black 96-well microtiter plates with transparent flat bottom (16503, Thermo Fisher Scientific, Rochester, NY, United States). OD_600_ and ROS were measured over 15 h at 27°C with a VICTOR Multilabel Plate Reader (Perkin Elmer, MA, United States). The outcome for ROS was measured in counts per second (CPS) when DCFH-DA was converted into 2′7′-dichlorofluorescein (DCF). All experiments consisted of 3–4 biological replicates.

### Filter Biofilms With Other Pathogens

To determine if other bacteria behaved in the same way as *P. aeruginosa*, we investigated the effect of anoxic conditioning on a selected group of pathogens (*S. epidermidis* ATCC 14990, *S. aureus* NCTC 8325-4 (methicillin susceptible), *S. aureus* USA300 JE2 (MRSA), *Escherichia coli* CFT073 and a clinical strain of *Enterococcus faecalis*). The filter biofilm method was used as described earlier. For the methicillin susceptible strain, CFU was determined in the same way as described for the filters with *P. aeruginosa*. For the remaining strains, CFU was determined at day 1 and 9 on LB plates supplemented with 10 mM NO_3_^–^ ± O_2_. Furthermore, CFU was determined on LB plates supplemented with 10 mM NO_3_^–^ and 0.3% sodium pyruvate to evaluate the effect of ROS. A minimum of 3 biological replicates was performed. CFU/mL was determined as previously described.

### Statistics

To evaluate the difference in bacterial growth between incubation conditions (normoxic and anoxic conditioning), a linear regression was used with the difference between the logarithmically transformed values for normoxic and anoxic plating as outcome and with day (categorical) and interaction between day and plating condition (binary) as explanatory variables in SAS Genmod Procedure. The *p*-value of the interaction term was used as the *p*-value for the difference in bacterial growth. Log difference was calculated as (log_10_[CFU/mL]_anoxic_ − log_10_[CFU/mL]_normoxic_). The mean and standard error of the mean (SEM) were calculated for recovering bacteria and plotted using GraphPad Prism 6.1 (GraphPad Software, La Jolla, United States). From the ratio between anoxic (CFU -O_2_) and normoxic (CFU + O_2_) colony counts it was possible to calculate the fraction of DTC bacteria $\left[\%\mathrm{DTC}\mathrm{bacteria}=\frac{\left(\text{CFU}-{\text{O}}_{2}\right)-\left(\text{CFU}+{\text{O}}_{2}\right)\,}{\left(\text{CFU}-{\text{O}}_{2}\right)\,}\times 100\right]$. Data that were not part of the long-term experiments were instead analyzed with a One-way ANOVA followed by Tukey’s multiple comparison tests or an unpaired *t*-test. A *p*-value ≤ 0.05 was considered statistically significant. The tests were performed with either Prism 6.1 (GraphPad Software, La Jolla, United States) or SAS v.9.4 (SAS Institute Inc., Cary, NC, United States).

## Results

### Anoxic Conditioning Generated Oxygen Intolerant Subpopulations of *P. aeruginosa* in Biofilm and Planktonic Models

**FIGURE 2:**
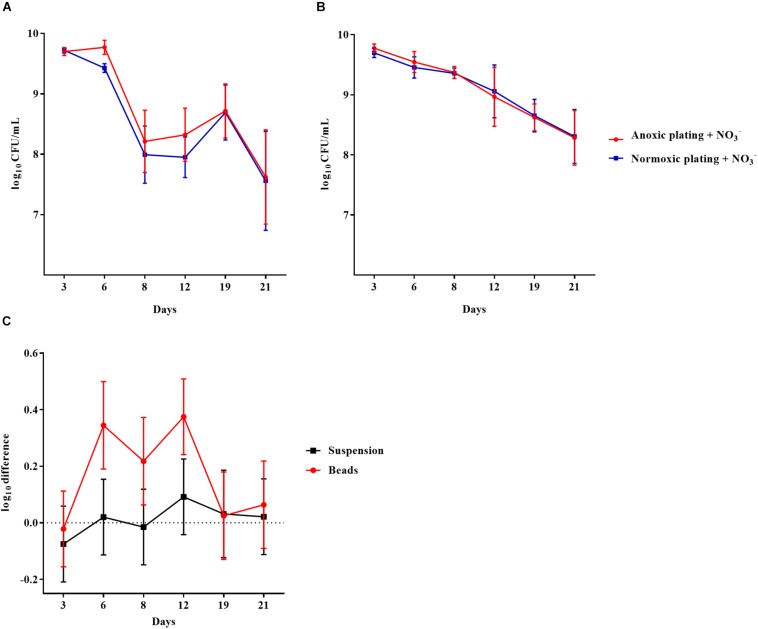
An oxygen intolerant subpopulation of *Pseudomonas aeruginosa* (PAO1) was generated in the bead biofilm model. Normoxic and anoxic colony forming units per milliliter (CFU/mL) of PAO1 over 21 days from the beads **(A)** and the surrounding suspension **(B)**. Symbols with error bars indicate the mean ± SEM (*n* = 4). + NO_3_^–^ refers to the addition of 10 mM KNO_3_ to LB agar plates. The log difference **(C)** represents the difference in mean log CFU/mL between plating methods from the suspension and the beads, respectively. The log difference was significantly higher (*p* = 0.003, linear regression) in the beads than the surrounding suspension. Symbols with error bars indicate the mean + confidence intervals.

To examine if biofilm growth affected the subsequent normoxic and anoxic plating, *P. aeruginosa* was grown in an alginate-bead biofilm model (Sønderholm et al., 2017). Plate counts were performed on LB plates supplemented with 10 mM NO_3_^–^. Plates were incubated under normoxic and anoxic conditions and plate counts were compared from the beads (biofilm) and the surrounding media (planktonic) of the same vials over a period of 21 days (Figures 2A,B, respectively). The log difference was significantly (*p* = 0.003) higher for biofilm than planktonic cells (Figure 2C). The cells represented by this difference were considered to be in a DTC state and ranged from 5 to 54% of the entire population from day 6 to 21 (Supplementary Figure S1).

**FIGURE 3:**
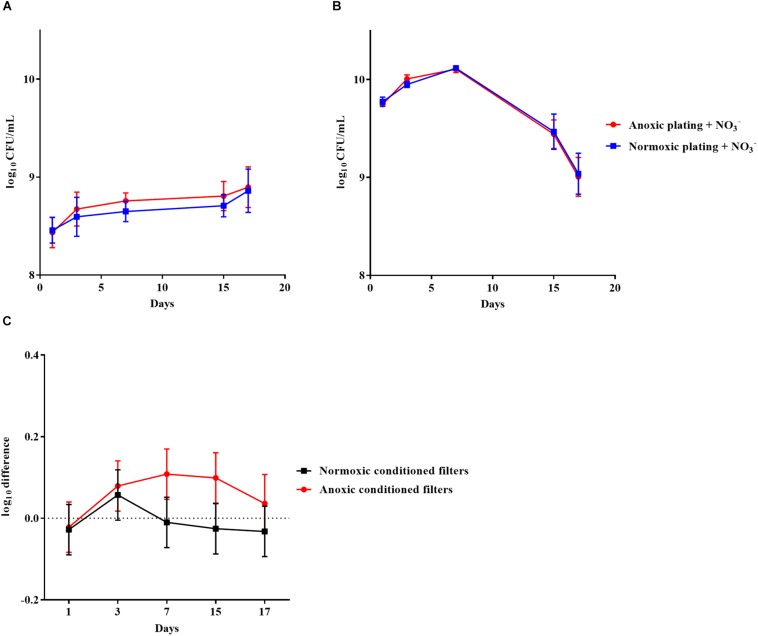
An oxygen intolerant subpopulation of *Pseudomonas aeruginosa* (PAO1) was generated in the filter biofilm model. Normoxic and anoxic colony forming units per milliliter (CFU/mL) of PAO1 over 17 days from anoxically **(A)** and normoxically **(B)** conditioned filters. Symbols with error bars indicate the mean ± SEM (*n* = 4). +NO_3_^–^ refers to the addition of 10 mM KNO_3_ to LB agar plates. The log difference **(C)** represents the difference in mean log CFU/mL between plating methods from the normoxically and anoxically conditioned filters, respectively. The log difference was significantly higher (*p* = 0.01, linear regression) in the anoxically conditioned filters than the normoxically conditioned. Symbols with error bars indicate the mean + confidence intervals.

*Pseudomonas aeruginosa* was also grown anoxically or normoxically on a filter biofilm model using LB plates supplemented with 10 mM NO_3_^–^ over a period of 17 days. CFU/mL was then determined using anoxic and normoxic plating, as above (Figures 3A,B, respectively). The log difference between plating methods was significantly (*p* = 0.01) higher for anoxically conditioned biofilms than normoxically conditioned biofilms (Figure 3C). The fraction of DTC *P. aeruginosa* ranged from 6 to 23% from day 3 to 17 (Supplementary Figure S1).

**FIGURE 4:**
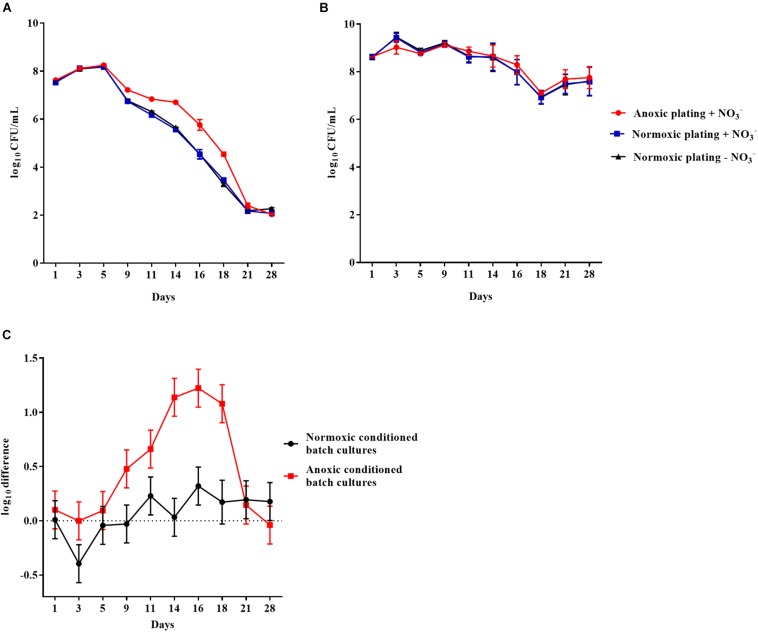
An oxygen intolerant subpopulation of *Pseudomonas aeruginosa* (PAO1) was generated in the planktonic batch cultures. Normoxic and anoxic colony forming units per milliliter (CFU/mL) of PAO1 over 28 days from anoxically **(A)** and normoxically **(B)** conditioned batch cultures. Symbols with error bars indicate the mean ± SEM (*n* = 4). ±NO_3_^–^ refers to the addition of 10 mM KNO_3_ to LB agar plates. The log difference **(C)** represents the difference in mean log CFU/mL between plating methods from the normoxically and anoxically conditioned batch cultures, respectively. The log difference was significantly higher (*p* < 0.0001, linear regression) in the anoxically conditioned batch cultures than the normoxically conditioned. Symbols with error bars indicate the mean + confidence intervals.

Similar results were obtained for anoxically and normoxically conditioned planktonic batch cultures of *P. aeruginosa* over a period of 28 days (Figures 4A,B, respectively). The log difference between plating methods was significantly (*p* < 0.0001) higher for anoxically conditioned batch cultures (Figure 4C). The fraction of DTC bacteria ranged from 11 to 93% from day 1 to 21 (Supplementary Figure S1).

**FIGURE 5:**
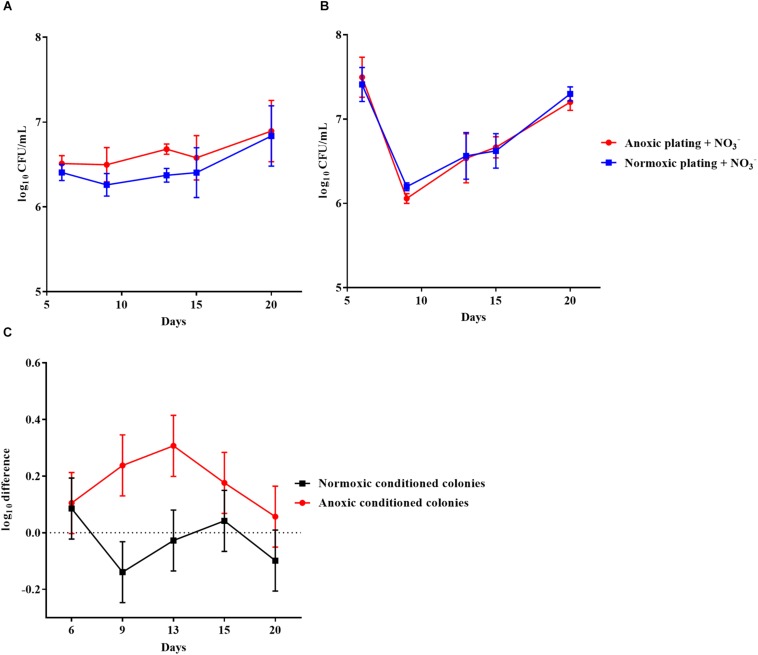
An oxygen intolerant subpopulation of *Pseudomonas aeruginosa* (PAO1) was generated in colonies. Normoxic and anoxic colony forming units per milliliter (CFU/mL) of PAO1 over 20 days from anoxically **(A)** and normoxically **(B)** conditioned colonies. Symbols with error bars indicate the mean ± SEM (*n* = 3). + NO_3_^–^ refers to the addition of 10 mM KNO_3_ to LB agar plates. The log difference **(C)** represents the difference in mean log CFU/mL between plating methods from the normoxically and anoxically conditioned colonies, respectively. The log difference was significantly higher (*p* < 0.0001, linear regression) in the anoxically conditioned colonies than the normoxically conditioned. Symbols with error bars indicate the mean + confidence intervals.

The effect of anoxic conditioning was further investigated on colonies of *P. aeruginosa* grown on LB plates supplemented with 10 mM NO_3_^–^ under anoxic and normoxic conditions over a period of 20 days (Figures 5A,B, respectively). The log difference between plating methods was significantly higher (*p* < 0.0001) for anoxically conditioned colonies (Figure 5C). The fraction of DTC *P. aeruginosa* ranged from 12 to 46% from day 6 to 20 (Supplementary Figure S1).

### 10 mM NO_3_^–^ in LB Plates Did Not Affect CFU/mL in Batch Cultures of *P. aeruginosa*

To ensure that presence of NO_3_^–^ did not affect the number of CFU generated on LB plates, CFU/mL was determined anoxically (LB plates + 10 mM NO_3_^–^) and normoxically (LB plates ± 10 mM NO_3_^–^) from 24-hour-old batch cultures of *P. aeruginosa*. No difference was observed between type of plating (*p* = 0.93): anoxic log CFU/mL + NO_3_^–^ = 9.96 (±0.15 SEM), normoxic log CFU/mL + NO_3_^–^ = 9.91 (±0.07 SEM) and normoxic log CFU/mL−NO_3_^–^ = 9.91 (±0.10 SEM). Furthermore, there was no effect of NO_3_^–^ supplementation on normoxic plating in the prolonged experiment with 28-day-old batch cultures (Figures 4A,B). It was not possible to detect growth of *P. aeruginosa* on LB plates under anoxic conditions without NO_3_^–^, but growth was observed when these plates subsequently were placed under normoxic conditions.

### Direct Viable Counts With LIVE/DEAD Staining Revealed a VBNC *P. aeruginosa* Population

**FIGURE 6:**
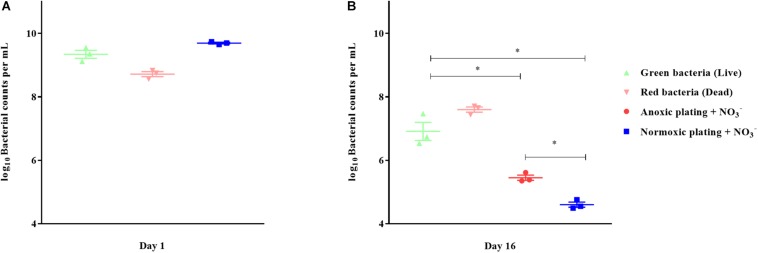
Direct viable counting reveals a viable but non-culturable population of *Pseudomonas aeruginosa* (PAO1) after anoxic conditioning. Bacterial counts per milliliter were determined with plate counting and direct viable counting from 24-hour-old batch cultures **(A)** and from 16-day-old anoxic conditioned batch cultures **(B)** of PAO1. Bacteria were stained with LIVE/DEAD staining to estimate the proportion of viable (live) and non-viable (dead) cells. There was significantly more viable bacterial counts when applying direct viable counting in comparison to normoxic plating (*p* < 0.0001, One-way ANOVA test) and anoxic plating (*p* = 0.0009, One-way ANOVA). Anoxic plating yielded significantly higher bacterial counts (*p* = 0.023, One-way ANOVA test) than normoxic plating. Symbols with error bars indicate the mean ± SEM (*n* = 3). + NO_3_^–^ refers to the addition of 10 mM KNO_3_ to LB agar plates. ^∗^*p* < 0.05.

Direct viable counts were carried out for 16-day-old, anoxically conditioned batch cultures of *P. aeruginosa* to investigate if anoxic conditioning could generate a VBNC population. Findings were compared to normoxic and anoxic plating (plating methods) performed on LB plates supplemented with 10 mM NO_3_^–^ (Figure 6B). Anoxic plating yielded a significantly (*p* = 0.023) higher count of CFU/mL than normoxic plating (log-difference = 0.85 log values ± 0.03 SEM). When comparing bacterial plate counts with direct viable counting, significantly (*p* < 0.0001) more viable cells were measured compared to normoxic plating (log-difference = 2.31 log values ± 0.33 SEM). This was also the case when comparing direct viable counting and anoxic plating (log-difference = 1.46 log values ± 0.35 SEM, *p* = 0.0009). The cells represented by the difference between direct viable counting and anoxic plating were considered VBNC. Direct viable counts were also performed on 24-hour-old batch cultures to investigate whether findings of VBNC *P. aeruginosa* were restricted to anoxically conditioned cells. No difference in bacterial counts was observed (Figure 6A).

### Oxidative Stress Restricted the Growth of Anoxically Conditioned *P. aeruginosa* When Re-Grown in a Normoxic Environment

**FIGURE 7:**
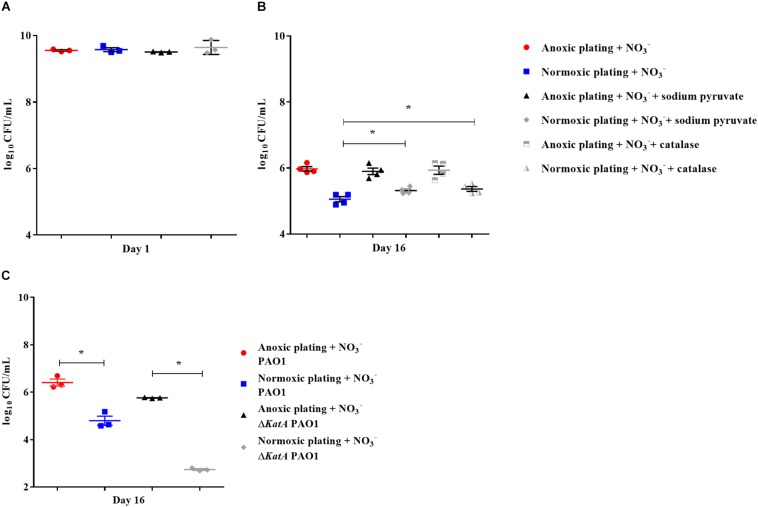
Reactive oxygen species generates a difficult-to-culture subpopulation of *Pseudomonas aeruginosa* (PAO1). Normoxic and anoxic colony forming units per milliliter (CFU/mL) were determined for 24-hour-old batch cultures **(A)** and 16-day-old anoxically conditioned batch cultures **(B)** of PAO1. Normoxic CFU/mL was determined ± 0.3% sodium pyruvate **(A,B)**, while addition of catalase was only tested for 16-day-old batch cultures. Symbols with error bars indicate the mean ± SEM (*n* = 4). + NO_3_^–^ refers to the addition of 10 mM KNO_3_ to LB agar plates. There was a significant difference between normoxic plating ± 0.3% sodium pyruvate (*p* = 0.04, One-way ANOVA test) and normoxic plating ± catalase (*p* = 0.03, One-way ANOVA test). Normoxic and anoxic determination of CFU/mL were determined for 16-day-old anoxically conditioned batch cultures of PAO1 and Δ*katA* PAO1 **(C)**. Symbols with error bars indicate the mean ± SEM (*n* = 3). Significant difference between anoxic and normoxic plating with both PAO1 and Δ*katA* PAO1 (*p* = 0.0014 and *p* < 0.0001, respectively, unpaired *T*-test). ^∗^*p* < 0.05.

**FIGURE 8:**
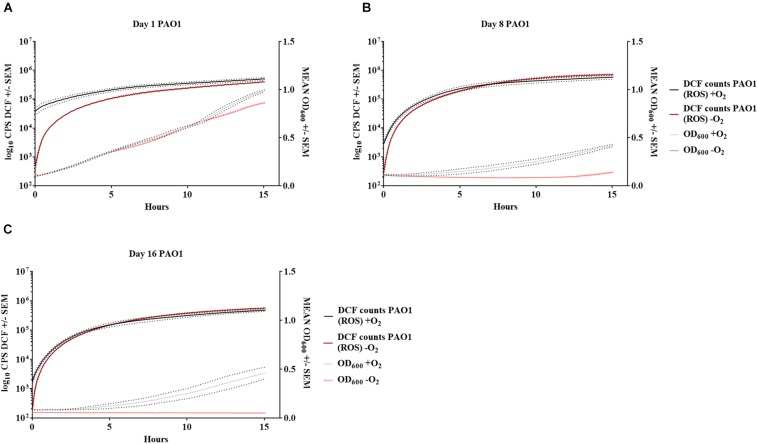
Increase in reactive oxygen species affects growth of anoxically conditioned *Pseudomonas aeruginosa* (PAO1) over time. Determination of reactive oxygen species (ROS) and optical density (OD) at day 1 **(A)**, 8 **(B)** and 16 **(C)** for anoxically (–O_2_) and normoxically (+O_2_) conditioned PAO1. ROS was measured as counts per second (CPS) when 2′,7′dichlorodihydrofluorescein diacetate was converted to 2′7′-dichlorofluorescein (DCF). Growth was measured with optical density (OD_600_) simultaneously to ROS measurement. Data is generated from 99 continuously measurements presented as a solid line with dots representing ± SEM (*n* = 4).

Bacteria from 16-day-old anoxically and normoxically conditioned batch cultures of *P. aeruginosa* were plated on 10 mM NO_3_^–^ LB plates ± sodium pyruvate or ± catalase to investigate whether formation of ROS contributed to the DTC state induced by anoxic conditioning. Presence of sodium pyruvate as a ROS scavenger (Mizunoe et al., 1999) significantly (*p* = 0.04) increased normoxic CFU/mL compared to plating without sodium pyruvate (Figure 7B). This was not the case in 24-hour-old batch cultures of *P. aeruginosa* (*p* = 0.62), indicating that the effect was restricted to anoxically conditioned cells (Figure 7A). Presence of catalase also significantly (*p* = 0.03) improved normoxic plate counts compared to normoxic plating without catalase (Figure 7B). These results show that hydrogen peroxide is involved in the induction of a DTC state created by anoxic conditioning. To add proof to this hypothesis, a similar experiment was carried out with a catalase deficient *P. aeruginosa* mutant (Δ*katA* PAO1), which is more susceptible to oxidative stress (Hassett et al., 1999; Jensen et al., 2014). The log difference between plating methods for the Δ*katA* mutant and reference strain was significantly (*p* = 0.00002, unpaired *t*-test) different. These log difference values were 3.02 ± 0.04 SEM and 1.61 ± 0.05 SEM for Δ*katA* PAO1 and the reference strain, respectively (Figure 7C). Moreover, measurement of ROS and OD was carried out for *P. aeruginosa* at day 1, 8, and 16 from anoxically and normoxically conditioned batch cultures. These results showed creation of ROS during normoxic growth. The longer PAO1 was anoxically conditioned; the lower was the following normoxic growth, as measured by OD. This effect was less pronounced for normoxically conditioned cells (Figures 8A–C).

### Anoxic Conditioning Affects *S. aureus* and *S. epidermidis*, but Not *E. coli* and *E. faecalis*

**FIGURE 9:**
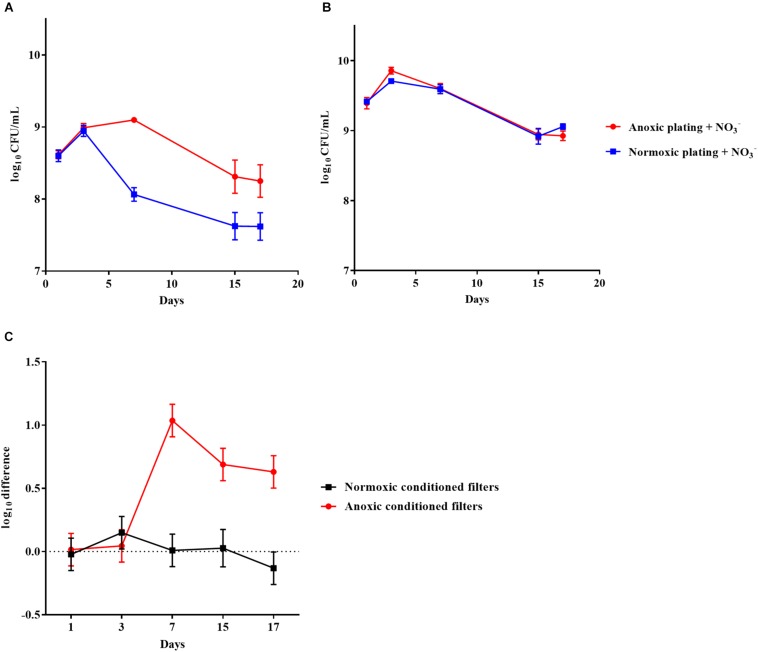
An oxygen intolerant subpopulation of *Staphylococcus aureus* (methicillin susceptible) was generated in the filter biofilm model. Normoxic and anoxic colony forming units per milliliter (CFU/mL) of *Staphylococcus aureus* over 17 days from anoxically **(A)** and normoxically **(B)** conditioned filters. Symbols with error bars indicate the mean ± SEM (*n* = 4). + NO_3_^–^ refers to the addition of 10 mM KNO_3_ to LB agar plates. The log difference **(C)** represents the difference in mean log CFU/mL between plating methods from the normoxically and anoxically conditioned filters, respectively. The log difference was significantly higher (*p* < 0.001, linear regression) in the anoxically conditioned filters than the normoxically conditioned. Symbols with error bars indicate the mean + confidence intervals.

The effect of anoxic conditioning was then tested on a selected group of pathogens to determine if this phenomenon was restricted to *P. aeruginosa*. *S. aureus* (methicillin susceptible) was tested as described in the *P. aeruginosa* filter biofilm setup. Plate counts for anoxically and normoxically conditioned filters was determined (Figures 9A,B, respectively). The log difference between plating method was significantly higher (*p* < 0.001) when plate counts were performed from anoxically conditioned filter biofilms than from normoxically conditioned filter biofilms (Figure 9C). The fraction of DTC *S. aureus* ranged from 3 to 89% from day 1 to day 17 (Supplementary Figure S1). Since both *P. aeruginosa* and *S. aureus* demonstrated effects of anoxic conditioning, a smaller experiment was initiated to test other pathogens. The effect was determined on LB plates supplemented with 10 mM NO_3_^–^ ± sodium pyruvate to investigate if ROS were involved in the lack of growth. Both *S. aureus* (MRSA) and *S. epidermidis* showed effects of anoxic conditioning, resulting in an significant (*p* = 0.01 and *p* = 0.03, respectively) increase in CFU/mL during anoxic plating compared to normoxic plating (Supplementary Figure S2). The fraction of DTC *S. aureus* (MRSA) and *S. epidermidis* was 36 and 42% at day 9, respectively. There was no observed effect of sodium pyruvate during normoxic plating for these two strains. In the case of *E. coli* and *E. faecalis*, there was no observed effect of anoxic conditioning (Supplementary Figure S3).

## Discussion

### DTC and VBNC *P. aeruginosa*

In the current study, we demonstrate that plate counting with *P. aeruginosa* is significantly affected by anoxic conditioning when cultured in the presence of atmospheric oxygen levels. A DTC state was created after only a few days of anoxic conditioning in both biofilm and planktonic models.

Difficult-to-culture *P. aeruginosa* was observed in the beads from the bead biofilm model, but not in the surrounding suspension, despite that the cultures had full access to atmospheric oxygen. This indicates that oxygen restriction within a biofilm generates a DTC subpopulation. The DTC state, detected as increased growth during anoxic plating with NO_3_^–^, has previously been generated in planktonic *P. aeruginosa* by energy starvation after cultivation without O_2_ as electron acceptor for aerobic respiration (Binnerup and Sørensen, 1993). *In vitro* biofilms of *P. aeruginosa* may contain internal anoxic zones (Walters et al., 2003; Kolpen et al., 2016), so we hypothesized that anoxia inside the biofilm contributes to the induction of DTC *P. aeruginosa*. Accordingly, DTC *P. aeruginosa* was only observed in the filter biofilm model and the colony model when the model was anoxically conditioned. Interestingly, we found that 99.68% of the population was VBNC with the LIVE/DEAD assay after anoxic conditioning in batch cultures. LIVE/DEAD staining is based upon membrane permeability and is only an approximation of true viability. Cells that were stained both red and green (resulting in yellow) were counted as dead cells but may still be viable. It would have been interesting to perform LIVE/DEAD staining on the biofilm populations in this study, but, due to the aggregation of bacteria in a biofilm, and the lack of proper method to dissolve these aggregates, we were concerned that a comparison against CFU would be biased.

Approximately 50% of the population in the alginate beads was DTC even though they were kept in normoxically conditioned vials. In comparison, the fraction of DTC cells was approximately 90% in the anoxically conditioned batch cultures. The difference (90 vs. 50%) may be explained by the fact that a majority of the bacteria in the beads are peripherally located where they have increased access to oxygen compared to the center of the beads (Sønderholm et al., 2017). In comparison, bacteria from the anoxically conditioned batch cultures were fully deprived of O_2_. In the present study, the effect of anoxic conditioning was restricted to an intermediate period. This is probably due to the static methodological setup. Dynamic processes, such as entry of nutrients and removal of waste, occur during infection and are not modeled here (Brown et al., 2008). Nevertheless, this is not the first time a “resuscitation window” has been described for non-culturable bacteria. Similar studies have shown that VBNC bacteria can only be detected in an intermediate period of culturing (Pinto et al., 2015). Besides the lack of dynamic processes, as mentioned above, these results are tested on a single strain of *P. aeruginosa*. In future studies it would be interesting to test whether this is a general trait for *P. aeruginosa* and if lack of culturing after anoxic conditioning is seen in various clinical strains. Lastly, it has to be mentioned that our results are based on only one type of media. It would be likewise interesting to test if other types of growth media produce similar results. Nevertheless, the authors of this paper feel confident in the results obtained.

### ROS Restricts Growth of Anoxically Conditioned *P. aeruginosa*

The DTC fraction of bacteria in this study was O_2_ intolerant given that growth only was achievable when NO_3_^–^ served as an alternative electron acceptor during anaerobic respiration. This led us to investigate whether this phenomenon was due to oxidizing properties of ROS created by incomplete reduction of O_2_ during aerobic respiration (Fenchel and Finlay, 2008). Sodium pyruvate increased counts for anoxically conditioned *P. aeruginosa* during normoxic plating (roughly 43% of the DTC population) and we believe that it was due to its properties as a ROS scavenger (Mizunoe et al., 1999). Accordingly, 0.3% sodium pyruvate has been used to resuscitate non-culturable populations of *S. aureus* (Pasquaroli et al., 2013). Catalase also increased counts during normoxic plating (roughly 50% of the DTC population), indicating that hydrogen peroxide is involved in the induction of a DTC state. The effect of ROS was further confirmed by the reduced growth of a catalase A deficient *P. aeruginosa* mutant (Δ*katA* PAO1) after anoxic conditioning. Finally, measurements of ROS along with OD showed that anoxically conditioned batch cultures of *P. aeruginosa* created more ROS when regrown under atmospheric conditions the longer they were deprived from oxygen, resulting in an accumulation of ROS and a reduction in growth represented by lower OD measurements. These results indicate that accumulation of ROS during aerobic respiration has an impact on normoxic plate counting when *P. aeruginosa* has been anoxically conditioned. Furthermore, it seems that hydrogen peroxide is involved in this loss of culturing. It has been suggested that VBNC cells cannot be resuscitated by addition of ROS scavengers and that “revived” cells in the presence of ROS scavengers are only injured cells and not VBNC cells (Pinto et al., 2015). It was not possible to determine whether DTC bacteria in this study were in an injured state.

### *S. epidermidis* and *S. aureus* Also Become DTC After Anoxic Conditioning

Additional experiments were conducted to elucidate whether anoxic conditioning generated DTC sub-populations in other facultative pathogens. Filter biofilms of methicillin susceptible *S. aureus* showed the same effects of anoxic conditioning as *P. aeruginosa*. These experiments were also repeated for *S. epidermidis*, *S. aureus* (MRSA), *E. coli* and *E. faecalis*. Both *S. epidermidis* and *S. aureus* were significantly (*p* = 0.01 and *p* = 0.03, respectively) affected by anoxic conditioning, whereas *E. coli* and *E. faecalis* were not. The results in this study points toward an intermediate period where anoxic conditioned bacteria are benefited by anoxic incubation with nitrate as alternative electron acceptor. We cannot rule out that we missed the period where *E. coli* and *E. faecalis* were DTC, given that they were tested at selected days. No effect of sodium pyruvate was found during normoxic plating for any of these tested organisms, suggesting that ROS is not involved in the loss of culturability for these pathogens, but additional ROS scavengers should be tested.

## Conclusion

A non-culturable state can be induced by several physiological stresses and our knowledge in this area is expanding, though far from fully resolved. In this study, we demonstrate that anoxic conditioning generates a VBNC subpopulation in *P. aeruginosa* batch-cultures. Furthermore, we demonstrate that anoxic conditioning generates DTC sub-populations of *P. aeruginosa*, *S. aureus*, and *S. epidermidis* during biofilm growth. The DTC population was only able to grow under anoxic conditions in the presence of NO_3_^–^ as an alternative electron acceptor. In the case of *P. aeruginosa*, this phenomenon was explained by creation of lethal amounts of ROS during aerobic respiration and the bacteria’s inability to neutralize it. Based on the results from this study, we suggest that a substrate with supplemented nitrate should be included during anoxic culturing of clinical samples from suspected biofilm infections.

## Data Availability

The data generated in this study are available from the corresponding author on reasonable request.

## Author Contributions

LK performed the majority of the experiments. MK, MS, BF, SC, and KK performed the experiments. TB, PJ, KK, AK, and LK conceived and designed the experiments. LK wrote the manuscript. MA and PJ performed the statistics. All authors analyzed the data, and contributed to and corrected the manuscript.

## Conflict of Interest Statement

The authors declare that the research was conducted in the absence of any commercial or financial relationships that could be construed as a potential conflict of interest.
