# Supplementary material for: Oxygen Restriction Generates Difficult-to-Culture P. aeruginosa
Source: Front Microbiol. 2019 Aug 29;10:1992. doi: 10.3389/fmicb.2019.01992 (PMC6727857; doi:10.3389/fmicb.2019.01992)
Supplement: Supplementary file 1 [file Data_Sheet_1.pdf]

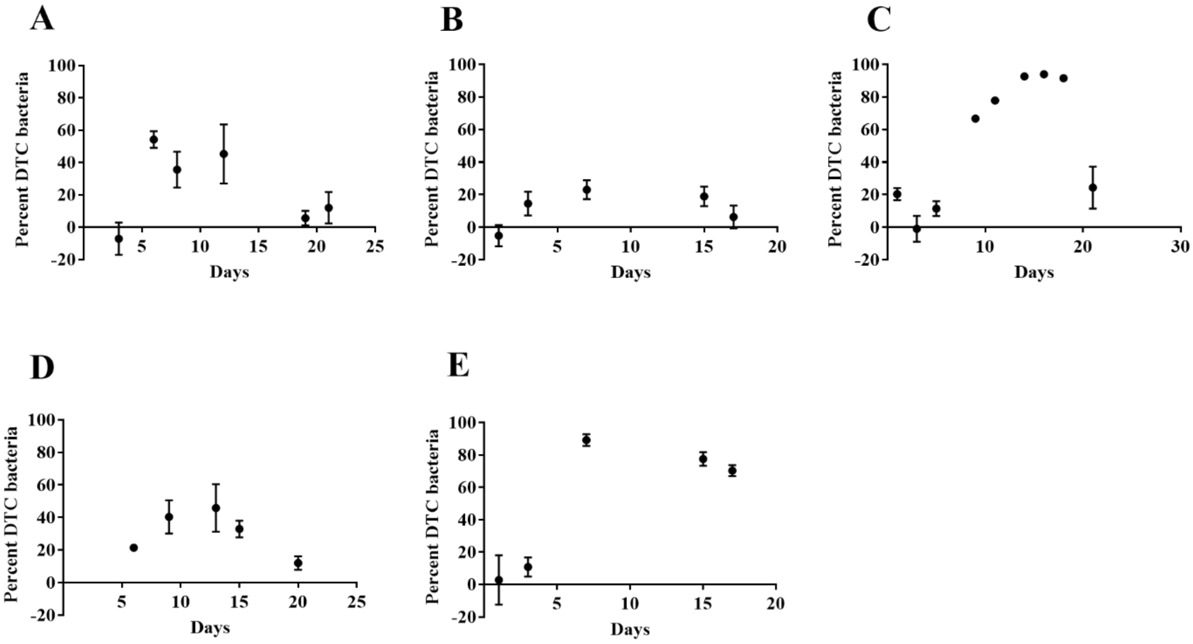

Fig. S1 – Percentage distribution of DTC bacteria over time in different growth models
The percentage of DTC *Pseudomonas aeruginosa* after anoxic conditioning in the bead biofilm model (A), filter biofilm model (B), batch cultures (C), colonies (D). Day 28 is missing in figure C because the value was lower than -20 % (-20.21 %). The percentage of DTC *Staphylococcus aureus* after anoxic conditioning in the filter biofilm model (E). Bars represent SEM. For some points, the error bars are shorter than the height of the symbol and therefore not displayed.
