# Supplementary material for: Oxygen Restriction Generates Difficult-to-Culture P. aeruginosa
Source: Front Microbiol. 2019 Aug 29;10:1992. doi: 10.3389/fmicb.2019.01992 (PMC6727857; doi:10.3389/fmicb.2019.01992)
Supplement: Supplementary file 2 [file Data_Sheet_2.pdf]

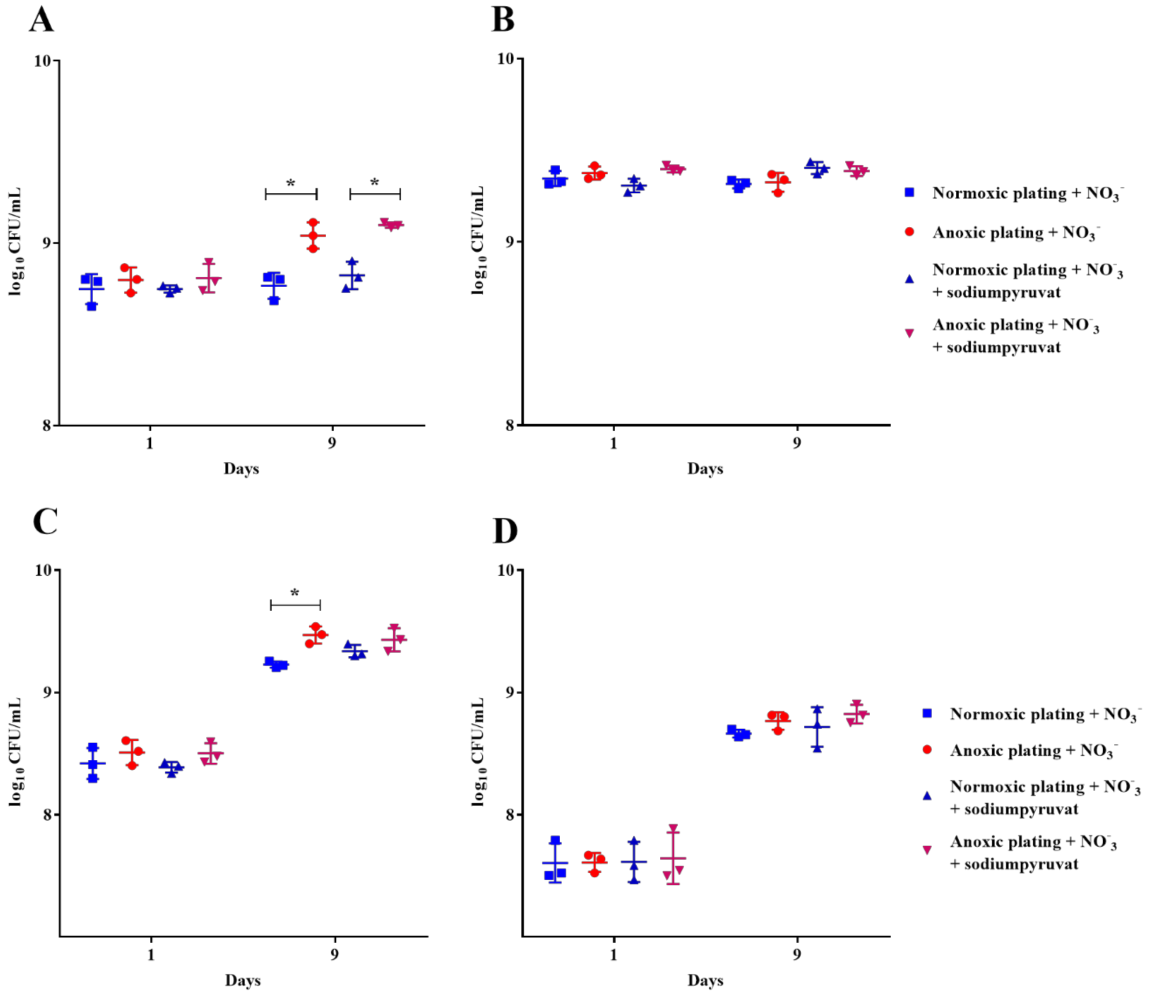

S2 - An oxygen intolerant subpopulation of S*taphylococcus aureus* (MRSA) and *Staphylococcus epidermidis* was generated in the filter biofilm model.
Normoxic and anoxic (± sodium pyruvate) determination of CFU/mL were determined for 1 and 9-day-old anoxically (A) and normoxically (B) conditioned filters with *Staphylococcus aureus* (MRSA) and anoxically (C) and normoxically (D) conditioned filters with *Staphylococcus epidermidis*. Significant difference between anoxic and normoxic plating ± sodium pyruvate at day 9 ($p = 0.01$ and $p < 0.01$, respectively, One-way ANOVA test) for *S. aureus*. Significant difference between anoxic and normoxic plating at day 9 ($p = 0.003$, One-way ANOVA test) for *S. epidermidis*. Symbols with error bars indicate the mean ± SEM (n = 3). $+NO_3^-$ refers to the addition of 10 mM KNO_3 to LB agar plates.
