# Supplementary material for: Oxygen Restriction Generates Difficult-to-Culture P. aeruginosa
Source: Front Microbiol. 2019 Aug 29;10:1992. doi: 10.3389/fmicb.2019.01992 (PMC6727857; doi:10.3389/fmicb.2019.01992)
Supplement: Supplementary file 3 [file Data_Sheet_3.pdf]

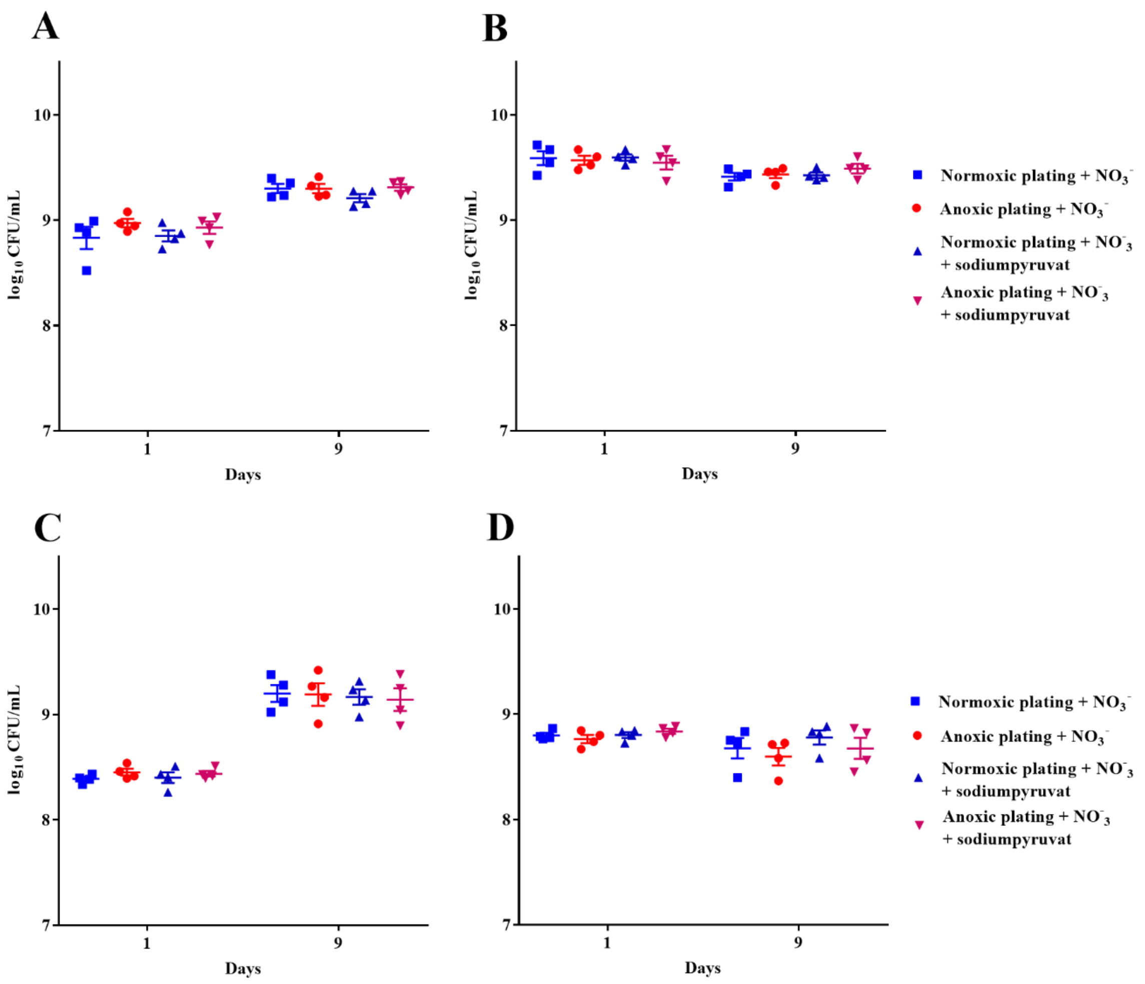

S3 – Anoxic conditioning did not affect E*scherichia coli* or *Enterococcus faecalis* during growth in the filter biofilm model.

Normoxic and anoxic (± sodium pyruvate) determination of CFU/mL were determined for 1- and 9-day-old anoxically (A) and normoxically (B) conditioned filters with *E. coli* and anoxically (C) and normoxically (D) conditioned filters with *E. faecalis*. No difference between types of plating (One-way ANOVA test). Symbols with error bars indicate the mean ± SEM (n = 4). +NO_3^- refers to the addition of 10 mM KNO_3 to LB agar plates.
